# Delayed alveolar bone repair and osteonecrosis associated with Zoledronic Acid therapy in rats: macroscopic, microscopic and molecular analysis

**DOI:** 10.1590/1678-7757-2020-0204

**Published:** 2020-09-25

**Authors:** Gustavo Zanna FERREIRA, Edson Virgílio ZEN, Izabel Regina Fisher RUBIRA-BULLEN, Gustavo Pompermaier GARLET, Carlos Ferreira SANTOS, Paulo Sérgio da Silva SANTOS

**Affiliations:** 1 UniCesumar Departamento de Odontologia MaringáPR Brasil UniCesumar - Departamento de Odontologia, Maringá, PR, Brasil; Universidade de São Paulo, Faculdade de Odontologia de Bauru, Departamento de Cirurgia, Estomatologia, Patologia e Radiologia, Bauru, SP, Brasil.; Universidade de São Paulo Faculdade de Odontologia de Bauru Departamento de Cirurgia, Estomatologia, Patologia e Radiologia BauruSP Brasil; 2 Clínica Odontológica Privada Brasil Clínica Odontológica Privada.; 3 Universidade de São Paulo Faculdade de Odontologia de Bauru Departamento de Cirurgia, Estomatologia, Patologia e Radiologia BauruSP Brasil Universidade de São Paulo, Faculdade de Odontologia de Bauru, Departamento de Cirurgia, Estomatologia, Patologia e Radiologia, Bauru, SP, Brasil.; 4 Universidade de São Paulo Faculdade de Odontologia de Bauru Departamento de Ciências Biológicas BauruSP Brasil Universidade de São Paulo, Faculdade de Odontologia de Bauru, Departamento de Ciências Biológicas, Bauru, SP, Brasil.

**Keywords:** Bisphosphonate-associated osteonecrosis of the jaw, Zoledronic acid, Bone regeneration, Disease models, animal

## Abstract

**Objective:**

This study aims to evaluate bone repair and the development of the medication related osteonecrosis of the jaw (MRONJ) associated with the use of zoledronic acid in Wistar rats.

**Methodology:**

48 male Wistar rats were divided into four groups: ZA, treated with intraperitoneal zoledronic acid, 0.6 mg/kg every 28 days, totaling five doses; control (C), treated with 0.9% sodium chloride; ZA-surgical (SZA) and C-surgical (SC), submitted to extraction of the right upper molars 45 days after the first application. Alveolar bone repair was evaluated by macroscopic and histological analysis. Protein expression evaluations were performed by qPCR.

**Results:**

Macroscopic evaluation showed that 91.66% (11) of the animals in the SZA group and 41.66% (5) from the SC group presented solution of epithelium continuity (P<0.05). All animals in the SZA group and none in the SC group had bone sequestration. The area of osteonecrosis was higher in the SZA group than in the SC group (P<0.05). In molecular evaluation, the SZA group presented changes in the expression of markers for osteoclasts, with increased RANK and RANKL, and a decrease in OPG.

**Conclusion:**

The results highlighted strong and evident interference of zoledronic acid in bone repair of the socket, causing osteonecrosis and delayed bone remodeling.

## Introduction

Bisphosphonates (BF) are synthetic drugs analogous to inorganic pyrophosphate, which are most commonly used in the treatment of bone disorders such as osteoporosis, bone metastases, Paget’s disease, and Multiple Myeloma.^1^ The first report describing bone exposure associated with the use of bisphosphonates was published in 2003.^2^ In 2014, the disease nomenclature changed to Medication-Related Osteonecrosis of the Jaw (MRONJ), as it was found that other anti-resorptive drugs and angiogenesis inhibitors may also cause bone exposure similar to that found in MRONJ, however bisphosphonates are still the major cause, mainly by the use of zoledronic acid.^3^

MRONJ pathophysiology is still unknown, however, risk factors for developing this condition can be classified into three categories: risk factors related to drug intake, local, and systemic risk factors.^4^ Among local risk factors, dentoalveolar surgeries,^5^ anatomical differences between maxilla and mandible; and preexisting oral pathology^3^ may be cited.

One of the possible pathways affected by BF is the RANK/RANKL/OPG system.^6^ RANK is a transmembrane receptor expressed on the surface of osteoclasts and osteoclasts precursor cells, and it is activated by the ligand RANKL, a protein typically produced by osteoblasts and T cells. In the presence of macrophage colony stimulating factor, RANKL stimulates the differentiation of osteoclast precursor cells into osteoclasts and also stimulates its activation, thus stimulating bone resorption. As a regulatory element of this system, OPG is a soluble receptor acting as “decoy” of RANKL, preventing the interaction between RANK and RANKL and bone resorption.^7-10^

Several attempts to develop animal models in rodents have already been published in the literature.^11,12,13-20,21,22,^ Therefore, this study applied an animal model under zoledronic acid, to study the effects of MRONJ on bone repair, after extraction of maxillary molar using microscopy and quantitative molecular analysis of mRNA expression involved in bone repair.

## Methodology

In this study 48 Wistar rats (*Rattus norvegicus albinus*) were divided into four groups (12 animals in each group), with 12 weeks of life. Due to hormonal variations, which may influence bone repair process, only male rats were used. Animals were feed with solid feed and water *ad libitum*. The study was approved by Research Ethics Committee (FOB-USP, CEEA 019/2011).

The rats were randomly divided into four groups:

- Control (C): animals received 0.9% sodium chloride in the same volume as the medications of the other groups and no tooth extractions.
- Zoledronic acid (ZA): animals received 0.6 mg/kg of zoledronic acid (Zometa^®^, Novartis) and no tooth extractions.
- Surgical control (SC): animals received 0.9% sodium chloride in the same volume as other medications and underwent extraction of right maxillary molars.
- Surgical zoledronic acid (SZA): animals received 0.6 mg/kg of zoledronic acid (Zometa^®^, Novartis) and underwent extraction of right maxillary molars.

**Figure 1:**
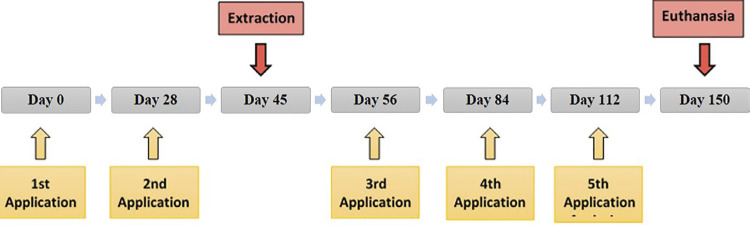
Outline of applications, surgical procedure, and euthanasia, related to time

Administration of the solutions was performed intraperitoneally, in the lower left quadrant of the abdomen, at 28 days intervals, totaling five doses, with the initial dose being given on the first day of the experiment (Figure 1). The dosage used in this experiment followed the model of Maahs, et al.^14^ (2011). Generally, intraperitoneal administration results in lower plasma concentrations than through intravenous route, and although the dose of 0.6 mg/kg may be considered slightly elevated, note that it was administered monthly, and its safety is supported by *in vivo* preclinical studies.^14^

As RT-PCR molecular analysis was performed in association with the histological analysis, we chose to use 12 rats in each group. Thus, we had a sufficient number of rats, six in each group for each analysis, to allow statistical analysis between the control and experimental groups.

### Surgical procedures

**Figure 2:**
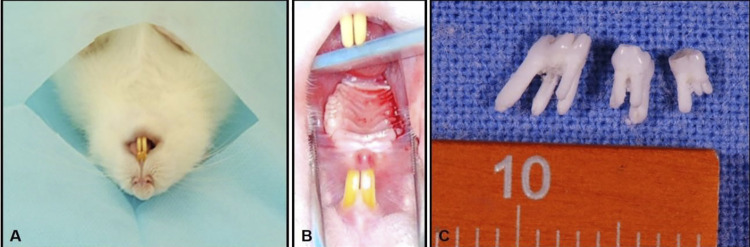
A) Positioning of the animal for maxillary molar extraction. B) Showing the region of the extracted maxillary teeth, with no need for suture. C) upper molars, extracted with intact roots

Forty-five days after the start of the experiment, the animals in the SC and SZA groups were submitted to the extraction of the first, second, and third maxillary right molars (Figure 2).

Surgical procedures were performed under general anesthesia with a combination of 25 mg/kg of ketamine hydrochloride and 10 mg/kg of xylazine hydrochloride administered intramuscularly. After surgical procedures, sockets were irrigated with 0.9% saline solution, and sutures were not necessary. Then, animals intramuscularly received ketoprofen for three days with dosage of 0.03 mL/100 grams of body weight, as postoperative analgesic. No antibiotics were administered pre and postoperatively

Prior to the surgical procedure, after anesthesia, an evaluator blinded to the experiment examined the presence or absence of lesions in the oral cavity. This analysis was repeated after euthanasia to identify the presence or absence of lesions, as well as to verify the presence or absence of oral mucosa solution of continuity in the extraction regions. The latter was performed with the aid of a #5 exploratory probe.^14^

Euthanasia was performed 150 days after the beginning of treatment, under sedation, by intramuscular injection of an excessive dosage of anesthetic.

### Microscopic analysis

The maxillae of the six rats of each group were fixed in 10% buffered formalin. The pieces were demineralized in aqueous solution of EDTA with pH 7.2. The blocks with the included maxilla were submitted to 4-μm thick semi-seriate cross sections that contemplated the post-extraction socket of the right maxillary molars and the region of preserved contralateral maxillary molars.

The histological images of the alveolar regions of molar extraction and regions of no molar extraction were captured using a digital analysis system, composed of Axioskop 2^®^ optical microscope mounted on AxioCam HRc^®^ and analyzed with the aid of AxioVision Rel. 4.8 Ink software^®^ using 40x and 100x magnification lens.

A specific area was delimited and standardized using the measurements made on the contralateral side of the maxilla to capture the images. As measurement reference, the palatine vascular-nerve bundle, 500 μm above the most apical region of the roots, and the most lateral and cervical portions of the alveolar bone ridge were used, thus defining a region of interest (ROI) of 3800 μm in width and 2500 μm in height.

**Figure 3:**
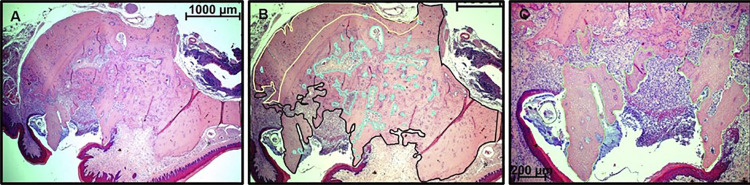
A, B, and C) Histological sections showing quantitative microscopic analysis of the region of interest (ROI) and the demarcation, in Figures B and C, of the areas to be measured. B) In yellow, the delimited periosteal reaction area; the black line is demarcating the bone tissue; and in blue, the areas of trabecular space. C) Shows the demarcation, in higher image magnification, of the areas with osteonecrosis and bone sequestration

The digitalized images were recorded in TIFF format, and a qualitative descriptive analysis was performed, as well as a quantitative analysis of the following variables: osteonecrosis, trabecular spaces, periosteal reaction, total bone, and presence of root residues in the extraction sites, with the aid of Axio-Vision Relay 4.8 Ink software^®^. These analyses were performed using the outline tool, enabling the delimitation of area of each evaluated variable, expressing the result in μm^2^. Root fragments and bone sequestration were evaluated for their presence or absence in the studied sections (Figure 3). Areas considered with osteonecrosis were the bone tissue regions in the alveolus that showed gaps for empty bone matrix lacunae (i.e. without osteocytes).^15^

### Molecular analysis

The right hemi-maxillae of the six animals of each group were cleaned, removing all soft tissue (gingiva, palatal mucosa, and periosteum) and they were stored in microcentrifuge tubes containing 1 mL of RNAlater Stabilization Reagent (Qiagen, Hilden, Germany). Tubes were agitated for 30 seconds, seated rest for 5 minutes, at room temperature, then frozen at -80°C. The process of RNA extraction followed the protocol of Kelly, et al.^23^ (2014).

The concentration of total RNA in the samples was determined by diluting RNA (known dilution factor, equal to 0.25) and absorbance reading of 2 μL of the sample in a Nanodrop ND-1000^®^, spectrophotometer at wave lengths of 260 nm (A_260_) and 280 nm (A_280_). The A_260_/ A_280_ ratio was estimated and results between 1.9 and 2.1 were considered acceptable, indicating absence of DNA in the samples.

RNA extracted from the samples was submitted to reverse transcription in cDNA with the Quantitect Reverse Transcription kit (Qiagen). The quantification of mRNA expression of alveolar bone repair factors OPG, RANK, and RANKL was performed by real-time PCR reactions (RealTimePCR) on a ViiA 7^®^ device, using the TaqMan system^®^. TaqMan primers/probes (Applied Biosystems) were used for the quantification of the target gene expression in each of the amplification reactions RANK (RN01426423_M1), RANKL (RN00589289_M1), OPG/TNFRSF11B (RN00563499_M1).

A negative sample was submitted to the reaction with each pair of sequences of the primers used. All samples were submitted to RNAm detection for RPL-13 (RN00821258_G1), which is a constitutive expression gene used as positive control of the amplification reaction. The results were analyzed based on the CT value (cycle threshold).

### Statistical analysis

The results of the macroscopic evaluation for the presence or absence of solution of continuity and the qualitative microscopic evaluation for the presence or absence of bone sequestration and root fragments in the region of dental socket of the SC and SZA groups were submitted to the Fischer’s exact test. For the results of quantitative microscopic analysis between the SC and SZA groups, unpaired Student t-test was applied for normal distribution, and Mann-Whitney test was used when the distribution was not normal. In the molecular analysis, the variables did not present a normal distribution and Kruskal-Wallis test was applied followed by Dunn post-test. The tests were performed using a software GraphPad Prisma 5^®^, 5% of significance level.

## RESULTS

All animals survived until the end of the experiment and their weights at day 0 and day 150 of the groups are shown in Table 1, and no statistically significant difference among groups and times was observed (P=0,323).

**Table 1:** Mean values and standard deviations (SD) of the animal weights (g) at day 0 and day 150 for C, ZA, SC and SZA groups

	C		ZA		SC		SZA	
	Day 0	Day 150	Day 0	Day 150	Day 0	Day 150	Day 0	Day 150
Mean	258.17	435.00	259.00	414.17	277.50	412.50	248.33	371.67
SD	17.51	18.97	14.56	42.12	39.08	58.72	38.82	44.12

ANOVA test: p=0.323

### Macroscopic evaluation

**Figure 4:**
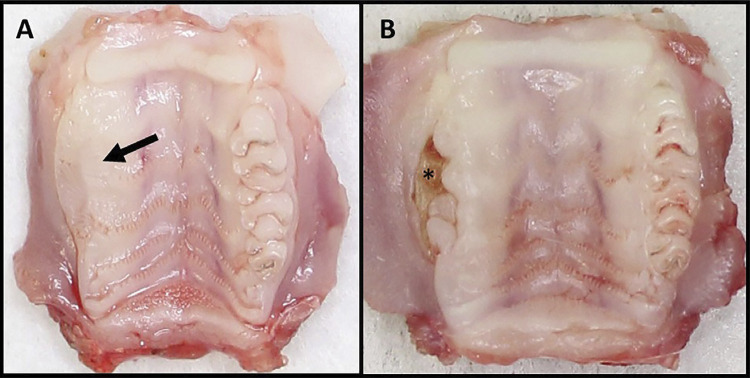
Jaws collected from rats showing repaired alveolus of: A) SC group, without solution of continuity (arrow); B) SZA group, with solution of continuity (*) of oral mucosa, with bone exposure area in the region of molar extraction

The intraoral evaluation performed in the animals at the days of medicine application, tooth extractions, and the day of euthanasia, did not show the presence of spontaneous lesions in any groups. On the other hand, the evaluation of the regions of maxillary molar extraction, at the day of euthanasia, showed a higher incidence of oral mucosal continuity in the animals from the SZA group (91.66%), compared with the SC group (41.66%) (P=0.0272) (Table 2, Figure 4).

**Table 2:** Presence/absence frequency of oral mucosal solution of continuity, bone sequestration and root fragments and in the socket area after maxillary molar extractions, in the SC and SZA groups

	Continuity solution #				Bone sequestration *				Root fragments			
Groups	Presence		Absence		Presence		Absence		Presence		Absence	
	n	%	n	%	n	%	n	%	n	%	n	%
SC	5	41.66	7	58.34	0	0	6*	100	6	100	0	0
SZA	11	91.66	1	8.34	6*	100	0	0	6	100	0	0

(*) Fischer exact test: p = 0.0022; (#) Fischer exact test: p = 0.0272.

### Qualitative microscopic analysis

The qualitative analysis of the maxillae in the teeth regions did not present relevant changes among the studied groups (C, ZA, SC and SZA). Most animals in the SC group presented bone repair with formation of mature bone tissue in the extraction site. All animals in SC and SZA groups presented root fragments in at least one studied region. Inflammatory infiltrate was usually found surrounding the root fragments.

In the SC group, when the site of tooth extraction presented oral mucosal epithelium continuity solution, this was, mostly, specific and related to the presence of root fragments. In the SZA group, solution of continuity was abundant and related to bone sequestration, which were frequent in this group. These characteristics are related to the findings in the macroscopic evaluation, in which the presence of a greater solution of continuity was observed in the SZA group.

**Figure 5:**
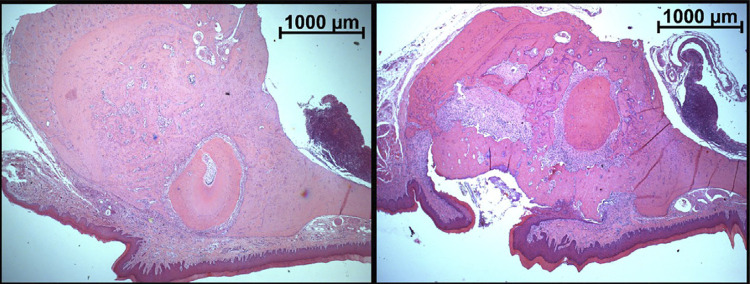
Histological section images of the first molar extraction site, from SC and SZA groups

The socket in the extraction sites of the SC group, in most cases, presented remodeling of the bone ridges and interradicular septa, with bone loss at the height of alveolar ridge. In the SZA group, a decrease in the loss of height of alveolar ridge, mainly due to a decrease in resorption of alveolar crests and interradicular septa was found. In some cases, bone sequestration occurred in the interradicular septa and alveolar bone crests. Bone sequestration often occurred in areas that presented aspect similar to segments of bone fracture, which may have occurred at the extraction (Figure 5).

### Quantitative microscopic analysis

In the microscopic analysis of the socket, bone sequestration was present in all animals from the SZA group (100%), whereas in the SC group no animals presented bone sequestration (0%) in the extraction area (p=0.0022) (Table 2).

**Figure 6:**
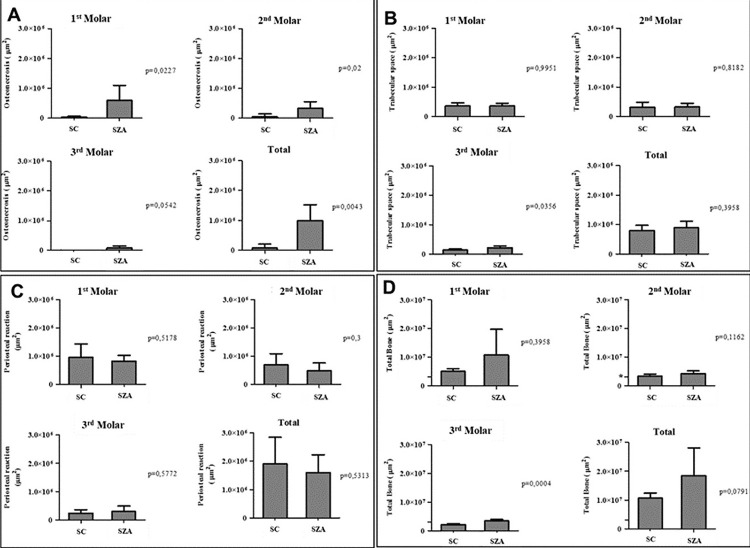
Graphs showing the areas of osteonecrosis (A), trabecular space (B), periosteal reaction (C), and total bone (D) in the regions of first, second, third molars and total area (sum of first, second, and third molars areas). The presented values represent means ± SD per group and area of each tooth. * Indicates the level 3.0 x 106 in the scale of total bone graphs, so that it can be compared with the graphs of the other studied variables. (*)= Kruskal-Wallis test

The results for osteonecrosis area were higher in the SZA group, with a statistically significant difference in the first and second molar regions (p=0.0227 and 0.02, respectively). Trabecular space and periosteal reaction variables did not present statistically significant difference between the groups SC and SZA. The total bone area, measured in each ROI, was higher in the animals of the SZA group. However, it presented a statistically significant difference only in the third molar region (p=0.0004) (Figure 6).

### Molecular analysis

**Figure 7:**
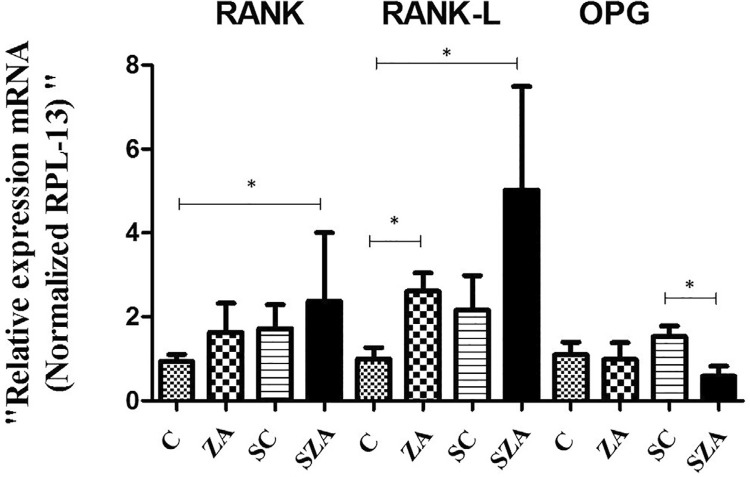
Expression of bone markers for levels of osteoclast expression for RANK, RANKL, and OPG, analyzed by qPCR. Results are presented as mean values ± SD of CT of the studied targets, normalized by RPL-13 expression, obtained from six animals of each group

RANK expression increased in the ZA, SC, and SZA groups, and there was a statistically significant difference between C and SZA groups (p=0.0481). In the RANKL variable, an increase in expression for the groups ZA, SC, and SZA was observed, presenting statistically significant difference between the groups C and ZA, C and SZA (p=0.0011). There was an increase in OPG expression in the SC group and a decrease in the SZA group, with statistically significant difference (p=0.0025) between both groups (Figure 7).

## Discussion

MRONJ is a serious complication that can affect patients undergoing therapy with BF. ^3^ This study used as a reference a model reporting the development of clinical and histological lesions in 100% and 80% of the animals, respectively.^14^ Although the clinical lesions of the tooth extraction region is slightly below 100%, it shows that this model^14^ was effective in the development of MRONJ-type lesions.

Osteonecrosis found in this study was higher than that observed in some experimental models that showed a high incidence of bone sequestration^14,16,20^. The areas of solution of continuity in the SZA group were greater, with large regions of bone tissue exposure and, when compared with histological findings, they were more related to areas of osteonecrosis and/or bone sequestration. These areas have been described in other studies evaluating the effects of BF on bone repair of dental socket in rats.^13,15,20^ Importantly, no formation of spontaneous lesions in the mouth was observed in any of the studied groups, which shows that dental extractions were significant factors in the development of MRONJ lesions.^14,20^

The sample collection was performed 105 days after extraction of the maxillary molars, therefore a complete repair of the socket would be expected,^24^ a fact that did not occur in the SZA group.

Most animals in SC group presented oral mucosa epithelium covering, corroborating previous studies results.^20^ The socket of animals in the SZA group presented lower loss in height and volume of the total area, compared with the SC group, with a decrease in the resorption of alveolar ridges and interradicular septa, and the formation of bone sequestration in most parts of these regions. This information may be corroborated by the fact that high dose of ZA administered over a long period of time may interfere in bone turnover time.^28^

Bone sequestration with preserved characteristics of the alveolar structure, indicating that no bone resorption of the alveolar ridges occurred four weeks after extraction of the maxillary molars in rats under ZA therapy, was also observed.^15,20^ Furthermore, this suggests that microfractures may have occurred during extractions, causing bone sequestration.^15^ Microfractures may also have occurred in the regions of interradicular septa and alveolar crests during extractions, which did not undergo reabsorption and remodeling, nor repair and consolidation.

A larger area of total bone in the socket was observed in the evaluated sections, with a trend for greater area of total bone in animals of the SZA group. This trend is possibly due to the lack of resorption and bone remodeling of the socket, observed in the qualitative analysis of histological sections. Other authors have demonstrated that tha analysis of alveolar bone remodeling showed minimal resorption after one week of tooth extraction in animals treated with zoledronic acid.^20^ Another study using the same dose of treatment with ZA was able to induce corticalization and decrease vascularization in the jaw.^29^

Regarding the molecular analysis, focused on the RANK/RANKL/OPG system, RANK and RANKL expression increased and OPG decrease in the animals in the SZA group, suggesting a greater activation of the osteoclasts.^30^ These results corroborate those found by Di Nisio, et al.^31^ (2015) who evaluated the expression of RANK, RANKL, and OPG in samples from patients treated with BF, who developed MRONJ. With these results, authors suggested a greater differentiation and activation of the osteoclasts, probably in viable areas of the necrotic bone, which could induce an increase in bone resorption. Moreover, OPG decreased, enabling the hypothesis of an induction of osteoclastogenesis. In this context, the activation of osteoclasts could be a protective mechanism of the bone tissue to delimit the necrotic area and eliminate infection.^31,32^ This is because the bacterial infection in the maxillae is one of the most likely hypotheses regarding the development of MRONJ.^32^

This elevation of RANK and RANKL is well demonstrated in studies on excessive orthodontic movement, with increased bone resorption.^33^ On the other hand, in some studies, rats that showed increased OPG expression had reduced bone resorption and developed severe osteopetrosis,^34^ whereas rats with decreased OPG expression showed severe osteoporosis due to bone resorption exceeding bone formation.^35,36^ There were similar results to those studies mimicking juvenile Paget’s disease in rats, where those rats with decreased OPG expression also presented elevated serum RANKL levels.^36^ These characteristics strengthen the hypothesis of osteoclastogenesis induction in this study.

Other authors^39^ demonstrated decreased RANKL expression and increased OPG in the mandible in rats (Sprague-Dawley) treated with zoledronic acid for 10 weeks, but without surgical manipulation. According to the authors, the increase in OPG levels in the mandible indicates osteoclastic inhibition. However, the decrease in RANKL levels in the mandible suggests a lack of osteoclasts activation. This change in RANKL levels with absence of osteoclastic activation could be a predisposing factor for developing MRONJ. However, the author in this model did not evaluate the incidence of MRONJ.

In our study, RANKL/OPG ratio of the SC group returned to values close to that of C group at the end of the experiment, corroborating with previous studies that evaluated the alveolar repair in Wistar rats in drug interventions and concluded that the favorable alveolar bone healing is expected at 28 days after tooth extraction in rats, with equilibrium between RANKL and OPG expression or even the predominance of OPG in the end.^40^ On the other hand, the administration of zoledronic acid increased the RANKL / OPG ratio mainly in the SZA group.^41^ One limitation of our study is that several attempts to develop animal models in rodents have already been published in the literature;^11,12,21,22,13-20^ however, each author applies different drug dosage, time of use and time between doses, extraction, and samples collection. Some researchers have used daily or weekly zoledronic acid doses for short periods of time, with samples collection being performed shortly after the administration of BF; others authors have used corticoids or immunosuppressive drugs^11,12,19,21^ in order to promote the antiresorptive action. Other co-factors used in murine ZA-treatment models are vitamin D deficiency,^15^ a drilled bone defect surrounding the dental extraction socket^13^ or mucosal lesion.^21,22^ Thus, it is not possible to compare the results obtained in our study with those found in other studies. In this study, the isolated effect of zoledronic acid administration was illustrated and the trauma of tooth extraction was a determining factor for the onset of MRONJ lesions, similarly to what other studies have shown,^14,20,21^ and it also evidenced the lesions by clinical, histological and molecular evaluation.

By molecular analysis, it was found divergent points in the expression of bone markers that may indicate differences in repair and consequently in the development of MRONJ. However, these markers were evaluated only in a late period of bone repair, which makes necessary further studies to evaluate the kinetics of bone remodeling, in established MRONJ models.
